# Endothelial dysfunction and its critical role in COVID‐19‐associated coagulopathy: Defibrotide as an endothelium‐protective, targeted therapy

**DOI:** 10.1002/jha2.198

**Published:** 2021-06-22

**Authors:** David García‐Bernal, Edward Richardson, Israel Vlodavsky, Carmelo Carlo‐Stella, Massimo Iacobelli, Rubén Jara, Paul G. Richardson, Jose M. Moraleda

**Affiliations:** ^1^ Department of Medicine Hematopoietic Transplant and Cellular Therapy Unit IMIB‐Arrixaca Virgen de la Arrixaca University Hospital University of Murcia Murcia Spain; ^2^ Frank H. Netter M.D. School of Medicine Quinnipiac University North Haven Connecticut USA; ^3^ Department of Surgery Yale University School of Medicine New Haven Connecticut USA; ^4^ Technion Integrated Cancer Center Rappaport Faculty of Medicine Technion Haifa Israel; ^5^ Department of Oncology and Hematology Humanitas Clinical and Research Center‐IRCCS Rozzano‐Milano Italy; ^6^ Department of Biomedical Sciences Humanitas University Rozzano‐Milano Italy; ^7^ Techitra s.r.l. Milan Italy; ^8^ Intensive Care Unit, Virgen de la Arrixaca University Hospital University of Murcia Murcia Spain; ^9^ Division of Hematologic Malignancy Department of Medical Oncology Dana Farber Cancer Institute Harvard Medical School Boston Massachusetts USA

To the Editor:

Recent publications emphasize that patients with COVID‐19 commonly present with lethal disseminated intravascular coagulopathy (DIC) characterized by increased levels of D‐dimer, fibrinogen, and elevated prothrombin time and activated partial thromboplastin time [1]. These abnormalities are associated with increased rates of thromboembolism and a profound prothrombotic state. However, other authors have found that both severe and mild COVID‐19 patients show similar levels of endogenous anticoagulants and similar anti‐thrombin, protein C, protein S, α2‐antiplasmin and plasminogen activator inhibitor‐1 (PAI‐1) activities [2], suggesting that we need new diagnostic criteria since the COVID‐19 coagulopathy is different to the usual forms of disseminated intravascular coagulopathy [3]. These authors conclude that preventive measures for thromboprophylaxis are key and diverse antithrombotic therapies may be especially helpful in COVID‐19 patients. It has been also reported that other markers of endothelial stress, including von Willebrand factor (VWF) and thrombomodulin, may predict mortality in COVID‐19 patients, suggesting therapeutic strategies to normalize endothelial cell function and protect vascular integrity are indeed vital [2].

The crucial role of endotheliitis in COVID‐19 has been substantiated by pathological findings from autopsies [4]. We wish to also highlight the possible role of heparan sulfate and heparanase in the endothelial dysfunction of COVID‐19, via the heparan sulfate‐heparanase pathway that triggers increased serum levels of inflammatory cytokines as part of disease pathobiology and additional targeted therapies derived thereon.

Specifically, SARS‐CoV‐2 damage to endothelial cells (EC), and subsequent endotheliitis [4] leads to upregulation of heparanase, an endo‐β‐glucuronidase that degrades the heparan sulfate scaffold of the glycocalyx and subendothelial basement membrane, increasing endothelial dysfunction and allowing extravasation of activated immune cells into the extravascular compartment [5, 6]. Heparan sulfate is an extracellular matrix sulfated glycosaminoglycan that protects mucosal epithelia and a co‐receptor for growth factors, cytokines, selectins, and viruses, including SARS‐CoV‐2. Additionally, heparanase is upregulated by pro‐inflammatory molecules and promotes expression of TNF‐α, IL‐6, MIP‐2, and IL‐1 in a pro‐inflammatory loop, which contributes further to inflammatory cytokines elevations [5].

Defibrotide is a complex mixture of poly‐deoxyribonucleotides extracted from porcine gut mucosa with pleiotropic properties, including anti‐thrombotic, pro‐fibrinolytic, anti‐inflammatory, and protective effects on small vessel endothelia [7]. Defibrotide increases tissue plasminogen activator and thrombomodulin expression, enhancing the activity of plasmin to hydrolyze fibrin clots, decreasing VWF, and PAI‐1. Platelet adhesion is inhibited via increased nitric oxide and prostaglandin E_2_ and I_2_ release. Conversely, defibrotide decreases inflammatory mediators including IL‐6, TNF‐α, VEGF, thromboxane A2, leukotriene B4, and reactive oxygen species. Defibrotide downregulates endothelial adhesion molecules such as P‐selectin, E‐selectin, ICAM‐1, VCAM‐1, and has been shown to inhibit leukocyte‐endothelial interactions [8]. Most importantly, defibrotide potently inhibits heparanase activity and its cell surface expression in variety of settings [7, 9]. Moreover, it blocks the heparanase‐heparan sulfate axis, by competing with heparan sulfate, and so may in turn inhibit both heparanase‐mediated viral release and spread, as well as heparanase‐mediated activation of immune cells and elevated inflammatory cytokines [5].

Defibrotide is approved for the treatment of severe veno‐occlusive disease/sinusoidal obstruction syndrome and has efficacy in patients with endothelial dysfunction and multiorgan failure, with activity in clinical studies and animal models of graft‐versus‐host disease incorporating lung injury [7, 8]. Its multitargeted endothelial‐based therapeutic properties make it a potentially ideal candidate to treat vascular complications of COVID‐19. Clinical studies are either planned or already underway in various countries, including Spain, Italy, and the United States, with the leading Spanish phase 2 study (ClinicalTrials.gov Identifier: NCT04348383) showing promising early results.

## RELATED LINKS

US National Library of Medicine. *ClinicalTrials.gov* https://clinicaltrials.gov/ct2/show/NCT04348383 (2020).

## AUTHOR CONTRIBUTIONS

All authors have contributed equally to this work.

## CONFLICT OF INTEREST

Dr. David Garcia Bernal, Dr. Israel Vlodavsky, and Dr. Massimo Iacobelli have no conflict of interest to declare. Dr. Carmelo Carlo‐Stella has received research support from ADC Therapeutics and Rhizen Pharmaceuticals; has served as consultant or advisor for Servier, Novartis, Genenta Science srl, ADC Therapeutics, Roche, Sanofi, Karyopharm and Jazz Pharmaceuticals; and has received honoraria for speaker engagements from Bristol‐Myers Squibb, Merck Sharp & Dohme, Janssen Oncology, and Astra‐Zeneca. Dr. Ruben Jara declares honorarium from Edwards Lifesciences and Getinge. Dr. Paul Richardson serves on advisory committees for Jazz Pharmaceuticals. Dr. Jose M. Moraleda reports grants from Jazz Pharmaceuticals, during the conduct of the study; consulting honoraria and travel expenses from Gilead, consulting honoraria from Novartis, Sandoz, and Takeda, outside the submitted work.
